# Hypoxia Promotes Invadosome Formation by Lung Fibroblasts

**DOI:** 10.3390/cells13131152

**Published:** 2024-07-06

**Authors:** Mégane Lebel, Dominic O. Cliche, Martine Charbonneau, Karine Brochu-Gaudreau, Damien Adam, Emmanuelle Brochiero, Claire M. Dubois, André M. Cantin

**Affiliations:** 1 Respiratory Division, Department of Medicine, Université de Sherbrooke, Sherbrooke, QC J1H 5N4, Canada; megane.lebel@usherbrooke.ca (M.L.); dominic.cliche@usherbrooke.ca (D.O.C.); andre.cantin@usherbrooke.ca (A.M.C.); 2 Department of Immunology and Cell Biology, Faculty of Medicine and Health Sciences, Université de Sherbrooke, 3001, 12^ième^ Avenue Nord, Sherbrooke, QC J1H 5N4, Canada; martine.charbonneau@usherbrooke.ca (M.C.); karine.brochu-gaudreau@usherbrooke.ca (K.B.-G.); 3 Centre de Recherche du Centre Hospitalier de l’Université de Montréal (CRCHUM), Montréal, QC H2X 0A9, Canada; damien.adam@umontreal.ca (D.A.); emmanuelle.brochiero@umontreal.ca (E.B.); 4 Department of Medicine, Université de Montréal, Montréal, QC H3T 1J4, Canada

**Keywords:** hypoxia 2, idiopathic pulmonary fibrosis, fibroblasts, invadosomes LPA, LPA_1_, nintedanib

## Abstract

Lung parenchymal hypoxia has emerged as a cardinal feature of idiopathic pulmonary fibrosis (IPF). Hypoxia promotes cancer cell invasion and metastasis through signaling that is dependent upon the lysophosphatidic acid (LPA) receptor, LPA_1_ (LPAR1). Abundant data indicate that LPA_1_-dependent signaling also enhances lung fibrogenesis in IPF. We recently reported that fibroblasts isolated from the lungs of individuals with IPF have an increased capacity to form subcellular matrix-degradative structures known as invadosomes, an event that correlates with the degree of lung fibrosis. We therefore hypothesized that hypoxia promotes invadosome formation in lung fibroblasts through LPA_1_-dependent signaling. Here, it is demonstrated that invadosome formation by fibroblasts from the lungs of individuals with advanced IPF is inhibited by both the tyrosine receptor kinase inhibitor nintedanib and inhibition of LPA_1_. In addition, exposure of normal human lung fibroblasts to either hypoxia or LPA increased their ability to form invadosomes. Mechanistically, the hypoxia-induced invadosome formation by lung fibroblasts was found to involve LPA_1_ and PDGFR-Akt signaling. We concluded that hypoxia increases the formation of invadosomes in lung fibroblasts through the LPA_1_ and PDGFR-Akt signaling axis, which represents a potential target for suppressing lung fibrosis.

## 1. Introduction

Idiopathic pulmonary fibrosis (IPF) is a chronic lung disease of unknown cause that is characterized by alveolar cell damage and the accumulation of activated fibroblasts leading to excessive interstitial matrix deposition and remodeling, which contributes to the reduced diffusing capacity of the lung [1]. Alveolar cells are unique in that they are exclusively perfused by deoxygenated blood, meaning that oxygen can only be supplied by diffusion from the airspace to alveolar cells, and not by diffusion from oxygenated blood as occurs in almost all other tissues [2]. Respiratory epithelial cells are particularly vulnerable to hypoxia if their surface is covered with fluid or mucin-rich secretions [3,4]. When alveolar or airway epithelial cells are submerged, access to oxygen decreases, thus stabilizing the hypoxia-induced transcription factor HIF-1, decreasing surfactant protein synthesis, and preventing epithelial cell differentiation, all of which can be reversed by supplemental oxygen [3,5,6]. Alveolar oxygen partial pressure (pAO_2_) is lower in the alveolar capillary units of the lung bases than in the upper lung zones, and pAO_2_ progressively decreases from the central to the peripheral airspaces [7,8]. These unique anatomical and physiological features render alveolar cells in the lower lung and sub-pleural zones, where increased matrix remodeling and fibrosis predominates, more susceptible to the perils of hypoxia.

Hypoxia has been documented in the lungs of humans with IPF and in animal models of pulmonary fibrosis [9,10,11,12]. The consequences of hypoxia include increased expression of the mucin Muc5B gene, which is associated with an increased risk of developing IPF [13,14]. The deposition of excessive amounts of mucin-rich secretions on the surface of alveolar cells has been documented in areas of fibroblast accumulation and extracellular matrix deposition in IPF lungs [14]. In addition, hypoxia increases the transcription of several pro-fibrotic genes, decreases surfactant synthesis, and enhances anaerobic glycolysis with the production of lactic acid, which favors the activation of TGFß, a key pro-fibrotic factor in the IPF lung [10]. In IPF fibroblasts, hypoxia has been reported to directly increase proliferation by reducing MNT, a c-myc inhibitor [15]. Chronic hypoxia has also been shown to promote the differentiation of lung fibroblasts into an activated myofibroblast like-phenotype, which may contribute to matrix deposition in the lung [16]. Taken together, these data underscore the importance of hypoxia in the initiation and maintenance of pulmonary fibrosis, but the cellular and molecular mechanism by which hypoxia contributes to the aggressive matrix remodeling phenotype of IPF fibroblasts remains incompletely understood.

An emerging player in the response to hypoxia is the LPA and LPA_1_ signaling cascade [17,18]. IPF is associated with a marked increase in lung LPA content, and several studies have documented the importance of the autotaxin-LPA_1_ axis in promoting pulmonary fibrosis [19,20,21]. Although the potential role of hypoxia in LPA_1_-dependent cell signaling in lung fibroblasts remains largely unknown, a recent study indicates that LPA led to the development of a pro-fibrotic phenotype in these cells. In addition, studies in cancer cells have clearly documented the importance of LPA and LPA_1_ during hypoxia in promoting the formation of aggressive actin- and metalloproteinase-rich invadosomes, which are subcellular structures involved in matrix proteolysis and metastasis [17,22]. Furthermore, we recently reported that fibroblasts derived from IPF lungs express a pro-destructive invadosomal phenotype, especially those from the most fibrotic areas of the lung [23]. The increased formation of invadosomes ex vivo was strongly correlated with the severity of lung fibrosis, providing further evidence of their role in fibrogenesis in vivo. This finding was consistent with a recent study showing that TKS5-depleted mice are resistant to bleomycin-induced lung fibrosis, which resulted in decreased invadosome formation in ex vivo cultures of lung fibroblasts [24]. Using invadosome assays involving mouse and human lung fibroblasts, we now report that hypoxia and LPA are potent inducers of invadosome formation. We also report that increased LPA_1_ and PDGFR-Akt signaling are likely to be part of the mechanism by which hypoxia promotes invadosome formation. These findings suggest that targeting LPA_1_ and PDGF-Akt signaling may represent a mechanistic rationale to limit lung fibrosis in IPF.

## 2. Materials and Methods

### 2.1. Reagents

Bleomycin was from the Hospira Healthcare Corporation (Lake Forest, IL, USA). Ketamine and Xylazine were from CDMV inc (St-Hyacinthe, Qc, Canada). Liberase TM of research grade, protein A agarose beads, complete™ Protease Inhibitor Cocktail, and PhosSTOP™ were obtained from Roche (Indianapolis, IN, USA). Nintedanib (cat#: S1010) was from Selleckchem (Huston, TX, USA). Gelatin from pig skin conjugated to Oregon Green 488, DAPI, and Texas Red™-X Phalloidin were from Invitrogen (Waltham, MA, USA). Pimonidazole and anti-pimonidazole (cat#: Pab2627) were from Hypoxyprobe inc (Burlington, MA, USA). CoCl_2_ (cat#: C8661-25G) and LPA (cat#: L7260-5MG) were purchased from Sigma (Oakville, ON, Canada). AM095 (cat#: A3166) was from APEXbio (Huston, TX, USA), and BMS-986020 (cat#: HY-100619) was from MCE (Monmouth Junction, NJ, USA). Autotaxin ELISA kit (cat#: LS-F16526) was from LS-bio (Lynnwood, WS, USA). Anti-PDGFRβ (cat#: 3169), anti-phosphorylated-PDGFRβ (cat#: 4549), anti-Akt (cat#: 9272), anti-phosphorylated-Akt (cat#: 9271), anti-β-actin (cat#: 4967S), and anti-rabbit HRP (cat#: 7074S) antibodies were from Cell Signaling (Whitby, ON, Canada). Anti-LPA_1_ antibody (cat#: ab23698) was from Abcam (Toronto, ON, Canada). RiboZol™ was purchased from VWR Life Sciences (Mississauga, ON, Canada). iScript™ supermix was obtained from Bio-Rad Laboratories (Mississauga, ON, Canada), and SYBR Green qPCR master mix was obtained from BiMake (Huston, TX, USA).

### 2.2. Experimental Mouse Model

All animal experimental procedures were performed in accordance with the animal use protocol approved by the Ethics Committee on Animal Research of the Université de Sherbrooke (CFPA-FMSS) and the guidelines of the Canadian Council on Animal Care. Eight to twelve-week-old C57BL/6 male mice were received from Charles River Laboratories. Mice were anesthetized with an intraperitoneal injection of ketamine/xylazine (87 mg/13 mg/kg body weight) and then exposed to bleomycin using a single intratracheal injection of 1 U/kg body weight. Lungs were collected after 28 days to perform fibroblast isolation and tissue fixation in formalin. For hypoxia detection studies, pimonidazole was administered at 60 mg/kg 2 h before lung harvest. Lungs from healthy mice were also collected and used as a control.

### 2.3. Immunohistochemistry

Lung tissues were fixed with 10% formalin and embedded in paraffin. Tissue sections of 4 µM were stained with Masson’s Trichrome by the Histology and Electronic Microscopy Platform at the Université de Sherbrooke. The following antibodies were used for immunohistochemical staining: Pab2627 antibody (pimonidazole detection) (dilution, 1:200); anti-CAIX (SantaCruz Biotechnology (Dallas, TX, USA), cat# sc-25599, dilution 1/50); anti-phospho-cortactin (Tyr421) (Cell Signaling, cat# 4569, dilution 1/50); and anti-cortactin (Millipore (Oakville, ON, Canada), cart# 05-180, dilution 1/50). Staining was revealed with 3,3′-diaminobenzidine (DAB; Agilent (Santa Clara, CA, USA), cat# K346711-2) and counter-stained with Harris hematoxylin. Microscope slides were scanned at 20X magnification with the Hamamatsu NanoZoomer 2.0 RS slide scanner (Hamamatsu Photonics, Bridgewater, NJ, USA) and at 5x magnification with a Zeiss AxioImager M2. For histological evaluations of mouse lungs, the presence of fibrosis and hypoxia was assessed following tissue staining with Masson’s trichrome and immunohistochemistry revealing pimonidazole, respectively. Assessment of fibrosis and hypoxia was based on sequential lung sections from two different lobes per mouse. Quantification of the pimonidazole-positive area was performed on 6 representatives images for each type of tissue using Image Pro software 6.0 (Media Cybernetics, Bethesda, MD, USA) according to the immunohistochemistry quantification technique described by Pham et al. [25].

### 2.4. Isolation and Culture of Lung Fibroblasts

Human lung tissue samples from healthy donors were obtained from the CRCHUM site, which is affiliated with the Quebec Respiratory Health research Network Biobank after obtaining written informed consent from patients prior to enrollment (protocol CER CHUM #08.063). This research study (#2018–2437) had the approval of the Université de Sherbrooke Institutional Review Board. Isolation of lung fibroblasts was performed as previously described [23]. The cells were cultured in DMEM supplemented with 15% fetal bovine serum, penicillin, streptomycin, and glutamine at 37 °C with 5% CO_2_. Additional normal human lung fibroblasts (NHLF #CC-2512) were purchased from Lonza and grown under the same culture conditions. These cells are designated primary human lung fibroblasts or p-HLF. In addition, lung fibroblasts from healthy mice and mice challenged with bleomycin for 28 days were similarly isolated and cultured. Fibroblasts from mice are labeled as primary mouse lung fibroblasts or p-MLF. Human and mouse cells were used between passages 3 and 7 at 70–80% confluency. In addition, the human HFL-1 cell line (fetal lung fibroblasts) was obtained from ATCC (CCL-153) and cultured in DMEM supplemented with 10% fetal bovine serum, penicillin, streptomycin, and glutamine. The HLF-1 cells were used between passages 3 and 9.

### 2.5. Invadosome Formation Assessment

The coverslips were coated with Oregon Green gelatin and prepared as described previously [23]. Lung fibroblasts were seeded on gelatin-coated coverslips with 0,1% FBS. After a 20–40 h incubation time, the cells were fixed with PFA (2%), permeabilized with saponin, and blocked with BSA (2%). Actin and nuclei were stained with Texas Red-X Phalloidin (dilution 1/200) and DAPI (0.1 µg/mL), respectively. Invadosomes were identified as actin-rich dots or dot-shaped areas at/or adjacent to areas of degraded gelatin using a Carl Zeiss fluorescence microscope (Carl Zeiss Inc., Thornwood, NY, USA), and images were captured at 40x magnification. Two coverslips were made for each experimental condition and 3 × 300 cells per coverslip were counted (total 6 × 300 cells) for the determination of the percentage of invadosome-forming cells. To quantify the number of invadosome structures per cell, fibroblasts were stained with anti-cortactin antibody, phalloidin-Texas red, and DAPI. Clusters of cortactin and actin were counted on micrographs from 20–25 cells, acquired using a scanning confocal microscope LSM Olympus FV1000 spectral (Olympus, Tokyo, Japan) with a 60× magnification. For experiments in which cells were incubated under hypoxic conditions, the cells (80% confluency) were serum-starved and placed in a humidified hypoxic chamber (In VivO_2_ 400 hypoxic workstation, Ruskinn, Bridgend, UK) at 1% O_2_, 5% CO_2_, and balanced in N_2_ for the period of time indicated in the figure legends.

### 2.6. mRNA Expression Analysis

Fibroblasts were grown to 80% confluency and then starved with serum-free DMEM for 16 h. Cells were incubated for 20 h in normoxia (21% O_2_) or hypoxia (1% O_2_) before RNA extraction (RiboZol™ reagent, VWR Life Sciences) according to manufacturer’s recommendations. RNA was reverse transcribed with iScript™ supermix using 0.5 µg per reaction. *SH3PXD2A* (TKS5), *CAIX* (carbonic anhydrase IX), *ENPP2* (autotaxin), *LPAR1* (lysophosphatidic acid receptor 1, LPA1), and *RPL13* transcripts (Table 1) were amplified by qPCR using SYBR Green qPCR master mix (BiMake) and Rotor-Gene 3000 (Corbett Research, Kirkland, QC, Canada). RPL13 was employed as the housekeeping gene control to calculate 2^−∆∆Ct^. The results are expressed as fold increase relative to the mean of the control condition.

### 2.7. RNAseq Data Analysis of Lung Tissue

Gene expression and clinical data from the GSE47460 dataset were obtained from the Gene Expression Omnibus database. Only data from healthy controls (N = 84) and IPF patients with measured % predicted DLCO (N = 110) were retained for this study. Fibrosis-, invadosome-, and hypoxia-associated genes were selected based on previous literature [26,27,28,29,30], and genes known to be downregulated in IPF [31,32] were used as negative controls. The relative gene expression for each selected gene was converted into z-scores prior to analysis. IPF patients were stratified according to IPF severity using % predicted DLCO (normal: ≥ 80%; mild: 40–79%; severe: < 40%). Modulation of gene expression with disease progression was assessed using ANOVA test coupled with Dunnett’s T3 multiple comparison test. Correlation of gene expression was assessed by two-tailed Spearman’s test.

### 2.8. Immunoblotting and Immunoprecipitation

Fibroblasts were grown to 80% confluence and then starved with serum-free DMEM for 16 h. Proteins were harvested using lysis buffer (Tris-HCl 50 mM, NaCl 150 mM, Na-deoxycholate 0.1%, EDTA 4 mM, NP-40 1%) containing phosphatase and protease inhibitors. Cell lysates (15–20 µg of total protein) were loaded onto 10% SDS-PAGE gels. Proteins were detected using a ChemiDoc MP Imager (Bio-Rad Laboratories). Band intensity was quantified using Image Lab software 6.0 (Bio-Rad Laboratories). For immunoprecipitation, anti-PDGFRβ antibody (dilution, 1:1000) was added to 200 µg of total protein before the addition of protein A agarose beads. Immunocomplexes were washed and loaded onto 10% SDS-PAGE gels. The separated proteins were then immunoblotted with the following antibodies: anti-PDGFRβ (dilution, 1:1000); anti-phosphorylated-PDGFRβ (pY857, pY751; dilution 1:1000); anti-Akt (dilution, 1:1000); anti-phosphorylated-Akt (pS473; dilution, 1:1000); anti-β-actin (dilution 1:1000); and anti-rabbit HRP (dilution, 1:5000) antibodies. For Western blotting of LPA_1_, the antibody was used a dilution of 1:500. Band intensities were analyzed using the Quantity One software 4.6.8 (Bio-Rad Laboratories, Richmond, CA, USA). Phosphorylation levels were determined after normalization, with the intensity of the band corresponding to the protein of interest. Expression levels were represented relative to the normoxic condition. Beta actin was also revealed on the same blot as the tested protein and used as a loading control.

### 2.9. Autotaxin Measurement by ELISA

Mouse lung fibroblasts were grown to 80% confluence and then starved with serum-free DMEM for 16 h. Cells were incubated for 40 h in normoxia (21% O_2_) or hypoxia (1% O_2_). After incubation, supernatants were collected to measure soluble autotaxin using the mouse ENPP2/autotaxin ELISA kit according to manufacturer’s recommendations. Fibroblasts were lysed, and the amount of cell protein was determined by the Bradford method to normalize the values. The concentration of autotaxin is expressed according to the following:

$$\frac{Autotaxin\;(ng)}{Supernatant\;(mL)}/Cell\;protein(mg)=Autotaxin\;levels.$$

### 2.10. Statistical Analyses

Statistics were performed using GraphPad Prism 8.01 software (GraphPad Software Inc. La Jolla, CA, USA). Each value (dot) in the histograms corresponds to data from a single human donor or a single mouse. The significance between two groups of data that were not normally distributed was determined by the unpaired Mann–Whitney test. For comparison of more than two groups, the Kruskal–Wallis test was used followed by Dunn’s multiple comparisons. The results obtained from the HFL-1 cell line were analyzed by Student’s *t* test (2 groups) or ANOVA test (more than 2 groups). Results are presented as mean ± SEM, and *p*-values are indicated as * *p* < 0.05, ** *p* < 0.01, *** *p* < 0.001, **** *p* < 0.0001. The abbreviation “ns” stands for not significant.

## 3. Results

### 3.1. Fibrotic Lungs of Mice Exposed to Bleomycin Exhibit Hypoxic Areas

**Figure 1:**
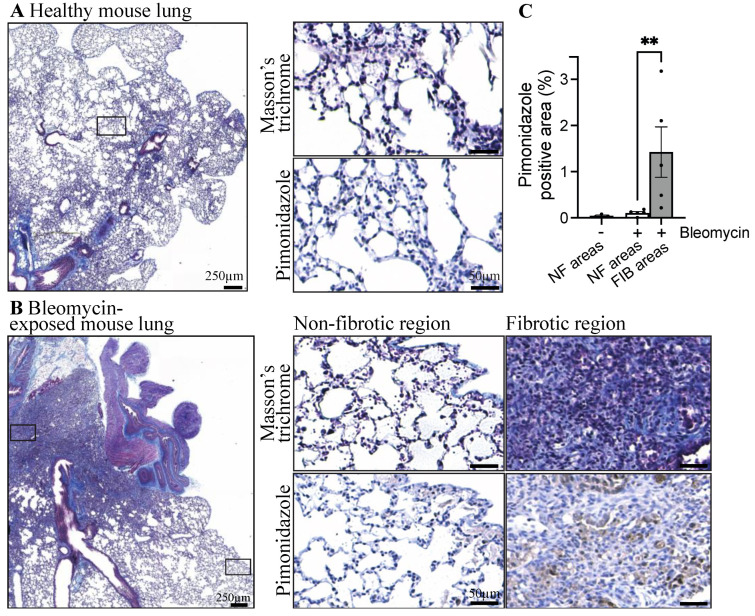
Hypoxic areas are found in the lungs of mice with pulmonary fibrosis. Images of lung tissue from (**A**) healthy and (**B**) bleomycin-exposed mice. Images are representative of three healthy and five bleomycin-exposed mice. Tissues are labeled for collagen (blue) via Masson’s trichrome staining and for hypoxia (brown) via immunohistochemistry using an anti-pimonidazole antibody. Higher magnification images show the architecture of the lung tissues. For the bleomycin-exposed mouse, two regions of interest were magnified, namely, a normal region and a fibrotic lesion, to visualize the presence of hypoxia and collagen. The scale of the highest magnification images is 50 µm. (**C**) Quantification of pimonidazole positive area staining in non-fibrotic (NF) areas of healthy lung tissues or in non-fibrotic (NF; *n* = 3) and fibrotic (FIB; *n* = 5) areas of bleomycin-exposed mice lung tissues. Bars represent the mean +/− SEM. ** *p* < 0.01, Mann–Whitney test.

To assess the presence of hypoxic areas in fibrotic lungs, pimonidazole was administered to healthy and bleomycin-exposed mice two hours before lung harvest. The presence of hypoxia and fibrosis were revealed via immunohistochemistry and Masson’s trichrome staining, respectively, on successive sections. The staining of these tissues revealed that healthy mouse lungs have undetectable levels of hypoxia (Figure 1). Bleomycin administration in mice results in heterogeneous lung fibrosis with dense fibrotic lesions adjacent to intact alveoli (Figure 1B, top image)**.** We then compared the presence of hypoxic regions in fibrotic *versus* normal subpleural regions. The results show that hypoxic areas were present in the lungs of mice exposed to bleomycin, particularly in the fibrotic and collagen-rich parenchyma (Figure 1B). Next, we evaluated the presence and location of hypoxia (pimonidazole staining) in lung sections from three healthy and five bleomycin-exposed mice. Hypoxia was virtually absent from non-fibrotic areas in both groups. However, fibrotic lesions in bleomycin-exposed mice showed a significant increase in areas positive for pimonidazole staining (Figure 1C). These observations confirmed the presence of hypoxia and its relationship with lung fibrosis in the bleomycin mouse model of pulmonary fibrosis.

### 3.2. Hypoxia Stimulates Invadosome Formation in Lung Fibroblasts

**Figure 2:**
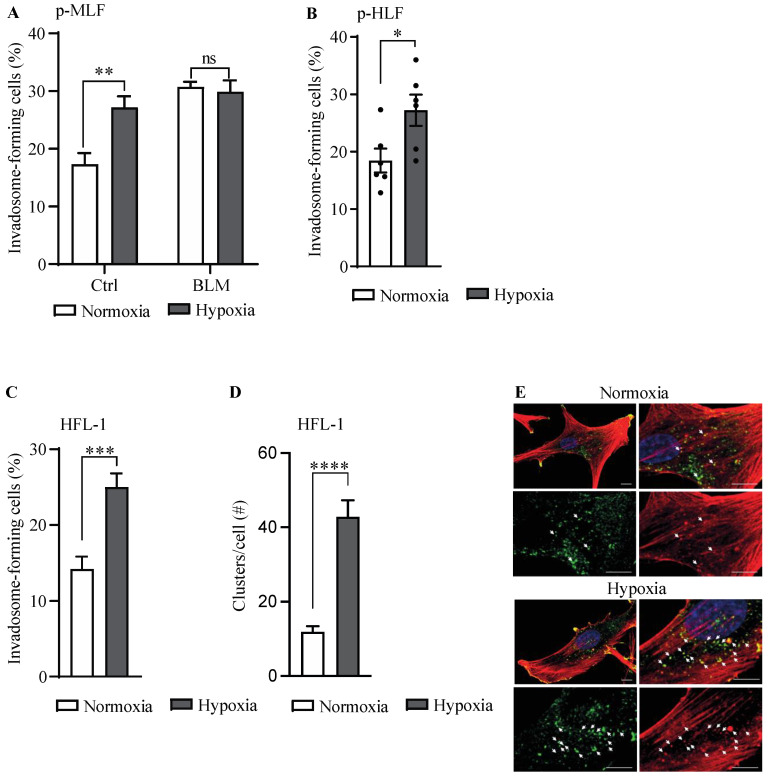
Hypoxia increased invadosome formation in lung fibroblasts. The percentage of invadosome-forming cells were assessed in lung fibroblasts cultured on fluorescent gelatin coverslips under normoxic or hypoxic conditions. Two coverslips were made for each experimental condition, and 3 × 300 cells per coverslip were counted (total 6 × 300 cells). (**A**) Primary mouse lung fibroblasts (p-MLF) isolated from healthy (Ctrl) (*n* = 6) or bleomycin (BLM)-exposed (*n* = 5) mice and cultured for 40 h in hypoxia; (**B**) primary human lung fibroblasts (p-HLF), where each dot represents a cell culture from one healthy individual. (*n* = 6) after 20 h of culture under hypoxia; and (**C**) HFL-1 cell line (*n* = 9). (**D**) The number of invadosomal structures identified through the colocalization of F-actin and cortactin was counted per cell (HFL-1 cell line). (**E**) Confocal micrographs of human fibroblasts stained for F-actin (red), cortactin (green), and nucleus (blue). Arrowheads show some representative colocalizations of F-actin and cortactin. Scale bar = 10 μm. For all experiments, data were obtained from at least three different mice or individuals in at least three independent experiments. Data are expressed as mean ± SEM. * = *p* < 0.05, ** = *p* < 0.01, *** = *p* < 0.001, **** = *p* < 0.001. ns = not significant.

Next, to investigate the impact of hypoxia on invadosome formation in lung fibroblasts, the percentage of invadosome-forming fibroblasts was evaluated following their incubation in normoxic or hypoxic conditions (Figure 2A). In fibroblasts from healthy mice, hypoxia augmented the formation of invadosomes by 1.6-fold, which corresponds to the level observed in fibroblasts from BLM lungs. However, in fibroblasts from BLM mice, hypoxia did not further modulate the percentage of invadosome-forming cells, which is consistent with their imprinted hypoxia-related signaling pathway [33]. To determine whether the modulation of invadosome formation by hypoxia also occurs in human lung fibroblasts, we performed invadosome assays with lung fibroblasts isolated from healthy donors (Figure 2B) and the human fetal lung fibroblast cell line HFL-1 (Figure 2C). The results showed that hypoxia increased the percentage of invadosome-forming cells by 1.5- and 1.8-fold in human lung fibroblasts and the HFL-1 cell line, respectively. As F-actin-enriched invadopodial core structures rely on cortactin for their maintenance, the presence of invadosomal structures was confirmed by the colocalization of F-actin and cortactin, which was 3-fold higher in hypoxic fibroblasts (Figure 2D,E). Taken together, these results suggested that hypoxia stimulates invadosome formation in fibroblasts from healthy lungs.

**Figure 3:**
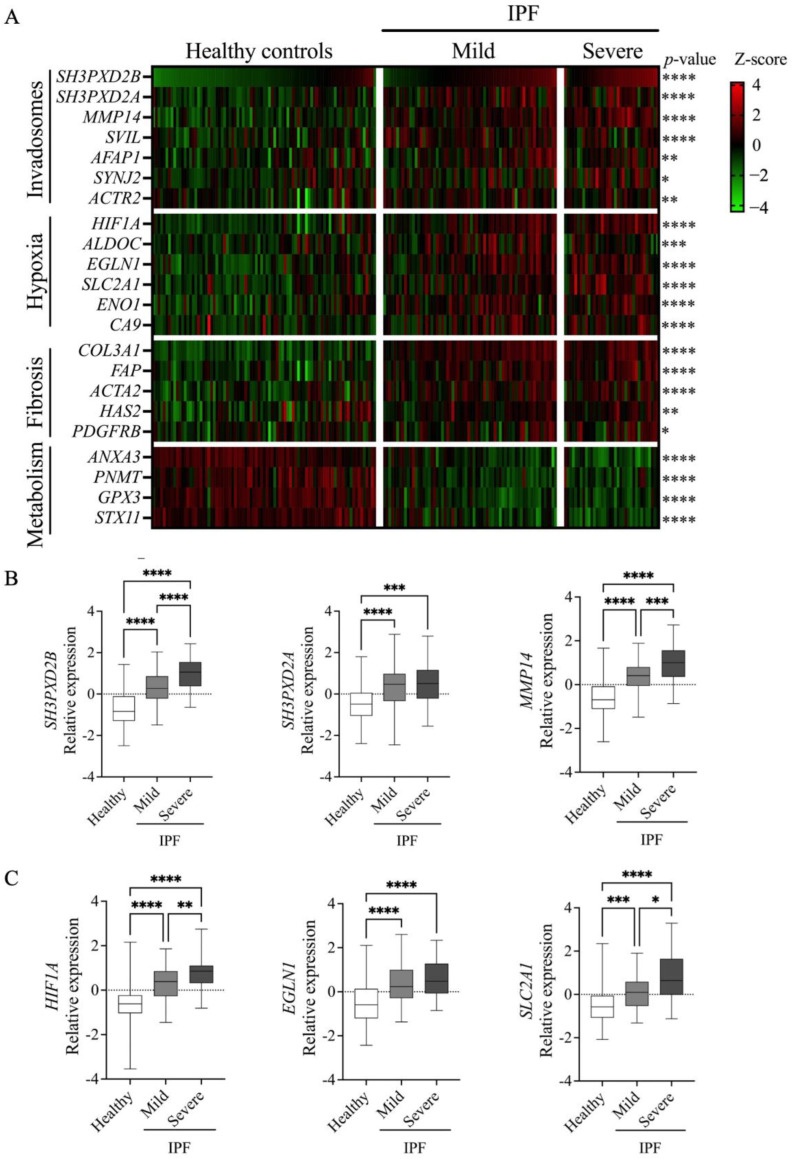
Hypoxia and invadosome-related gene expression in healthy controls and IPF patients of increasing disease severity: (**A**) heatmap illustrating the relative expression of each gene for each healthy control (N = 84) and IPF patient presenting mild (N = 67) or severe (N = 35) % predicted DLCO; (**B**,**C**) bar graphs illustrating the relative expression of invadosome (**B**) or hypoxia-associated (**C**) genes for healthy controls (N = 84) and IPF patients presenting mild (N = 67) or severe (N = 35) % predicted DLCO. * *p* < 0.05, ** *p* < 0.01, *** *p* < 0.001, **** *p* < 0.0001.

**Figure 4:**
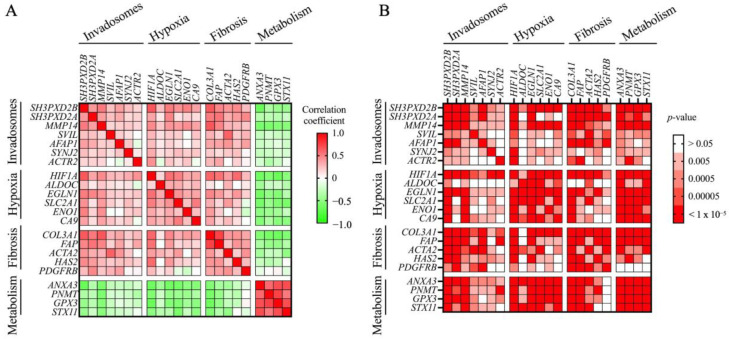
Invadosome, hypoxia, fibrosis, and metabolism-related gene expression correlation in lung tissues from IPF patient cohort GSE47460: (**A**) heatmap illustrating Spearman correlation coefficient of selected gene pairs; (**B**) heatmap illustrating associated *p*-values for the correlations presented in (**A**).

**Figure 5:**
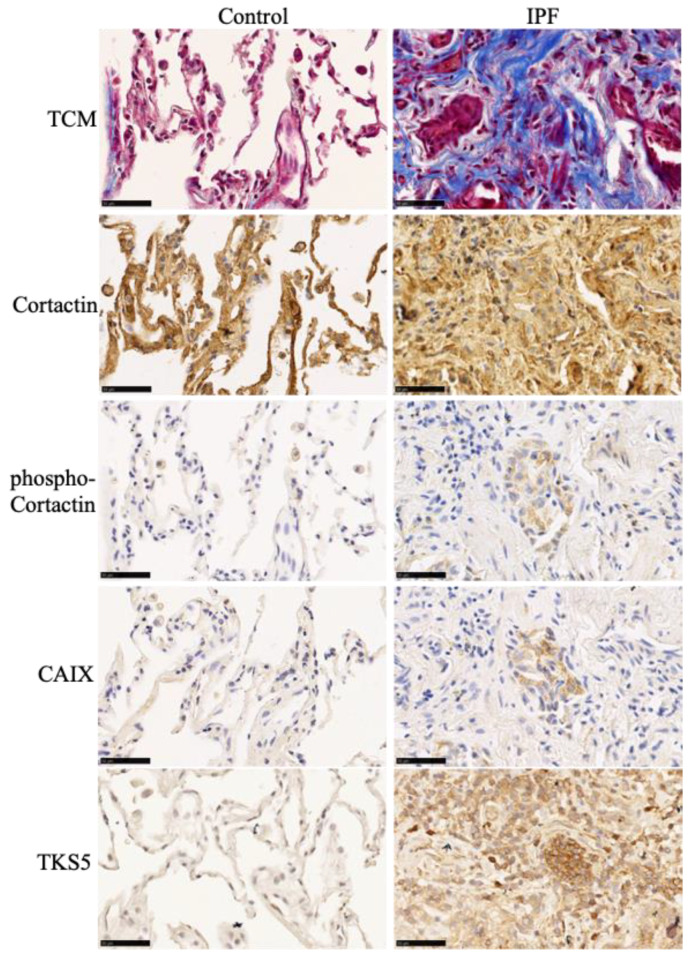
Invadosome markers are present in hypoxic collagen-rich areas of IPF lungs. Representative images of lung tissue from healthy and IPF patients. Tissues are labeled for collagen (blue) via Masson’s trichrome staining and for cortactin, phospho-cortactin, CAIX, or TKS5 (brown) via immunohistochemistry. N = 4; scale bar = 50 μm.

Using publicly available gene expression datasets, we have previously shown that key genes involved in invadosome formation are expressed in IPF lung tissue samples, particularly in regions corresponding to fibroblast/ECM-rich foci, reminiscent of invadosome formation by fibroblasts in fibrotic areas of the lung [23]. Using a similar approach, we now asked whether IPF lungs have a genetic profile associated with hypoxia and whether this profile correlates with an invadosomal signature. The results show a significant increase in the hypoxia-associated genes (*HIF1A*, *ALDOC*, *EGLN1*, *SLC2A1*, *ENO1*, *CA9* 6/19/24 3:53:00 PM); fibrosis-associated genes (*COL3A1*, *FAP*, *HAS2*, *PDGFRB*); and genes linked to invadosome formation (*SH3PXD2B*, *SH3PXD2A*, *MMP14*, *SVIL*, *AFAP1*, *SYNJ2*, *ACTR2*) [28,29,30] in IPF patients with increasing disease severity compared to healthy subjects (Figure 3A–C). In comparison, the metabolism-related genes *GPX3, ANXA3*, *PNMT*, *GPX3*, and *STX11* are downregulated, as previously reported [31,32]. Furthermore, most invadosome-related genes were significantly positively correlated with those involved in hypoxia or fibrosis, whereas genes associated with cell metabolism were inversely correlated (Figure 4A,B). To further investigate the presence of invadosomes in hypoxic and fibrotic zones of IPF tissues, we performed immunochemistry staining for the activated form of cortactin (p(Tyr421)) and TKS5, two invadosome markers that have been shown to localize to actin-rich invadopodial structures and to correlate with invadopodia activity [34,35]. The results, presented in Figure 5, show that the activated form of cortactin (pTyr421) and TKS5 are present in the CAIX-positive (a marker of hypoxia), collagen-rich areas of IPF lungs, further suggesting the relevance of invadosome formation during in vivo fibrogenesis.

### 3.3. The Effect of Lysophosphatidic Acid Receptor 1 (LPA_1_) Activation on Invadosome Formation in Lung Fibroblasts

**Figure 6:**
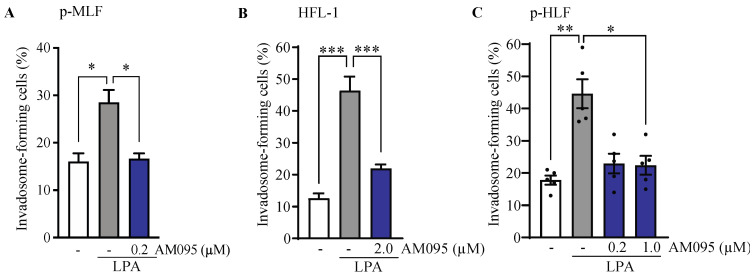
LPA increases invadosome formation in healthy lung fibroblasts through LPA_1_. The percentage of invadosome-forming cells were assessed in lung fibroblasts cultured on fluorescent gelatin-coated coverslips in the presence or absence of LPA and the LPA_1_ antagonist AM095 used at the indicated concentrations. Two coverslips were made for each experimental condition, and 3 × 300 cells per coverslip were counted (total 6 × 300 cells). (**A**) Primary mouse lung fibroblasts (p-MLF) isolated from healthy mice after 40 h in hypoxia; (**B**) HFL-1 cell line and (**C**) primary human lung fibroblasts (p-HLF) after 20 h in hypoxia. Each dot represents a cell culture from one healthy individual. *n* = 5. For all experiments, data were obtained from at least three different mice or individuals in at least three independent experiments. Data are expressed as mean ± SEM. * = *p* < 0.05, ** = *p* < 0.01, *** = *p* < 0.001.

In various cancer cell lines, hypoxia is known to promote the formation of invadosomes by potentiating LPA-LPA_1_ signaling [22]. Given that LPA_1_ is a target of interest in the pathophysiology and treatment of IPF [36], we sought to determine whether LPA_1_ activation could promote the formation of invadosome in lung fibroblasts. The results showed that the addition of LPA increased invadosome formation by 1.8-fold in mouse lung fibroblasts (pMLF) (Figure 6A) by 3.5-fold in HFL-1 cells (Figure 6B) and by 2.5-fold in primary human lung fibroblasts (p-HLF) (Figure 6C). Interestingly, the addition of the LPA_1_ antagonist, AM095 [37], blocked LPA-stimulated invadosome formation in each cell type. These results demonstrated that LPA_1_ is essential for the LPA-mediated invadosome formation in lung fibroblasts.

### 3.4. Hypoxia Implicates LPA_1_ and Receptor Tyrosine Kinases (RTK) for Invadosome Formation in Lung Fibroblasts

**Figure 7:**
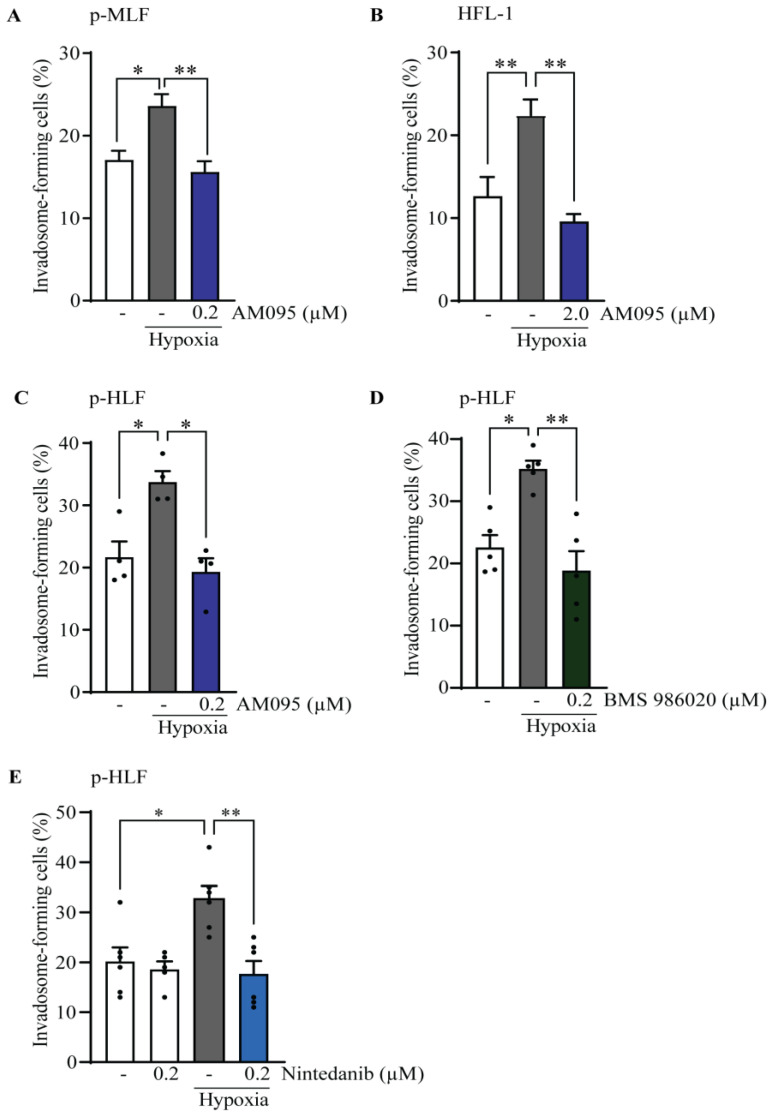
Hypoxia increased invadosome formation in healthy lung fibroblasts through LPA_1_ and RTK. The percentage of invadosome-forming cells were assessed in lung fibroblasts cultured on fluorescent gelatin-coated coverslips in normoxia or hypoxia and in the presence or absence of LPA_1_ antagonists (AM095 or BMS-986020) or nintedanib. Two coverslips were made for each experimental condition, and 3 × 300 cells per coverslip were counted (total 6 × 300 cells). (**A**) Primary mouse lung fibroblasts (p-MLF) isolated from healthy mice after 40 h under hypoxia; (**B**) HFL-1 cell line; and (**C**–**E**) primary human lung fibroblasts (p-HLF). Each dot represents a cell culture from one healthy individual (*n* = 4–5) after 20 h under hypoxia. For all experiments, data were obtained from at least three different mice or individuals in at least three independent experiments. Data are expressed as mean ± SEM. * = *p* < 0.05 and ** = *p* < 0.0.

Given that hypoxia promotes LPA-LPA_1_ signaling [22], an event involved in lung fibrosis [38], we tested whether the LPA_1_ receptor was required for hypoxia-stimulated invadosome formation. The results demonstrate that the addition of AM095 prevents the formation of hypoxia-stimulated invadosomes in fibroblasts isolated from mouse lungs (Figure 7A), in HFL-1 cell line (Figure 7B), and in fibroblasts isolated from human lungs (Figure 7C). These results were further confirmed in human lung fibroblasts using a second LPA_1_ antagonist: BMS-986020 [36] (Figure 7D). Furthermore, no changes were detected in the mRNA and protein levels of LPA1 and autotaxin (Figure S1) under hypoxic conditions, indicating that the mechanism of action is likely post-transcriptional.

Since hypoxia has been shown to promote RTK and LPA_1_ crosstalk leading to invadosome formation [22], we further investigated whether nintedanib, an inhibitor of RTKs, including PDGFR, FGFR, and VEGFR, could affect invadosome formation under hypoxia. The results showed that 0.2 µM nintedanib was sufficient to block the effect of hypoxia on invadosome formation in primary human lung fibroblasts (Figure 7E). These results suggest the importance of LPA_1_ and RTKs in invadosome formation by lung fibroblasts under hypoxic conditions.

### 3.5. LPA_1_ Is Involved in Hypoxic Stimulation of PDGFRβ Phosphorylation

**Figure 8:**
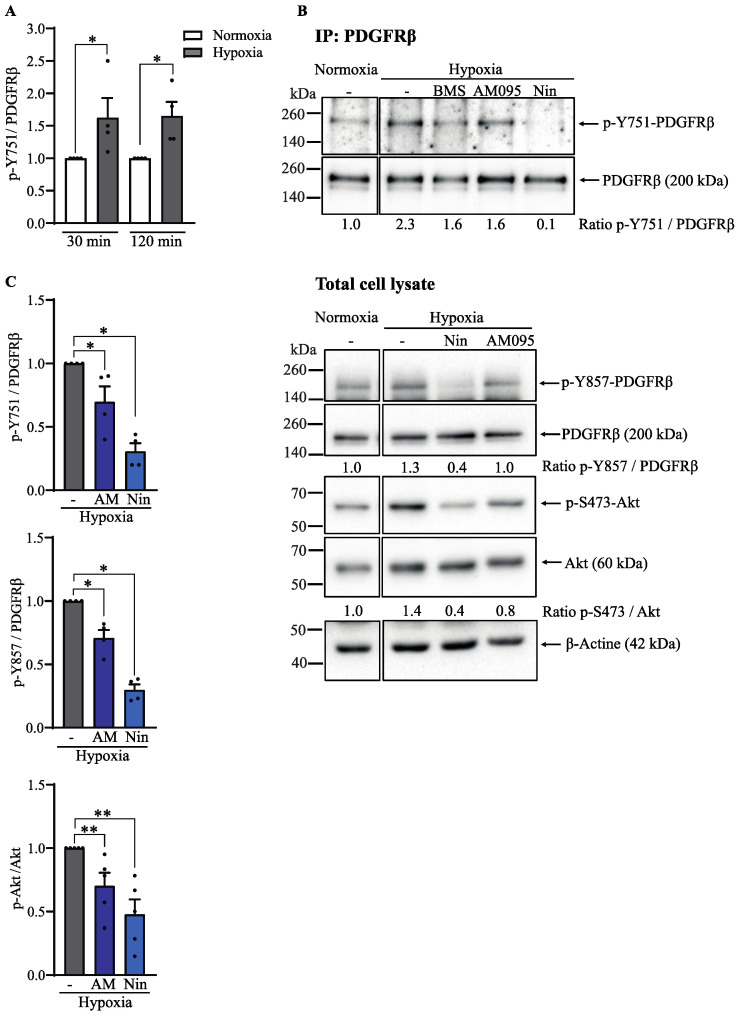
LPA_1_ was involved in the activation of PDGFRβ and Akt under hypoxia in healthy lung fibroblasts. Primary human lung fibroblasts were incubated under normoxia or hypoxia in the presence or absence of nintedanib 0.2 µM, AM095 0.2 µM, or BMS-986020 0.2 µM. Immunoblots of total cell lysates. (**A**) Cells were incubated under hypoxia for 30 and 120 min. Densitometric analysis is relative to normoxia. (**B**) Representative experiment of 4–5 independent experiments involving PDGFRβ immunoprecipitation (IP:PDGFRβ) followed by immunoblotting for phosphorylated PDGFR (Y751) and total PDGFRβ with corresponding ratios relative to hypoxia. Below, immunoblots of total cell lysates showing phosphorylated PDGFR (Y857), total PDGFRβ, phosphorylated Akt (S473), and total Akt with corresponding ratio relative to normoxia. (**C**) Immunoblots of total cell lysates showing phosphorylated PDGFR (Y857), total PDGFRβ, phosphorylated Akt (S473), and total Akt with corresponding ratio relative to normoxia. Corresponding densitometric analysis relative to hypoxia of four to five independent experiments. Each dot represents a cell culture from one healthy individual. Data are expressed as mean ± SEM. * = *p* < 0.05 and ** = *p* < 0.01.

In a previous study, we demonstrated that nintedanib blocked invadosome formation in healthy lung fibroblasts stimulated with PDGF-BB [23]. Given the importance of LPA_1_ and RTKs (PDGFR, FGFR, and VEGFR) for invadosome formation under hypoxic conditions, we sought to determine whether LPA_1_ is involved in the hypoxic activation of PDGFR. PDGFRβ phosphorylation at Y751, a PI3K binding site, was increased by 1.6- and 1.7-fold in cells exposed to hypoxia for 30 and 120 min, respectively (Figure 8A). Furthermore, PDGFRβ immunoprecipitation results showed that the increased phosphorylation of Y751 induced by hypoxia was strongly inhibited by the addition of nintedanib and partially inhibited by the LPA_1_ antagonists AM095 and BMS-986020 (Figure 8B). Using total cell lysate extracts, we also showed that the levels of phosphorylated PDGFRβ at Y857 were increased by 1.3-fold in hypoxia, which was inhibited by nintedanib or AM095. Similarly, hypoxia induced a 1.4-fold increase in Akt phosphorylation, which was prevented by nintedanib or AM095 (Figure 8B). Results from densitometric analysis of 4–5 independent experiments showed that the addition of AM095 to hypoxia-exposed cells reduced the phosphorylation status of both PDGFRβ sites and Akt by 30%. As expected, the presence of nintedanib also impaired the levels of phosphorylated PDGFRβ and Akt by 70% and 52%, respectively (Figure 8C). Taken together, these results suggest that hypoxia promotes invadosome formation in lung fibroblasts, likely through potentiation of LPA_1_ and PDGFR-Akt signaling.

## 4. Discussion

Multiple studies have confirmed the presence of hypoxic areas in the lungs of mice with fibrosis [39], and hypoxia has emerged as a cardinal feature of IPF lungs [9,40]. In the context of cancer cells, the hypoxic environment has been found to stimulate the formation of invadosomes, specialized structures involved in extravasation and metastasis [41]. Recently, we have identified invadosome structures in mouse and human lung fibroblasts [23]. Furthermore, we have shown that fibroblasts isolated from IPF and bleomycin-exposed mouse lungs had an increased ability to form invadosomes compared to healthy fibroblasts [23]. Here, we report that hypoxia induces invadosome formation in primary lung fibroblasts, a process blocked by the antifibrotic drug nintedanib and LPA_1_ antagonists. We also report that the effect of hypoxia likely occurs through potentiation of LPA_1_ and PDGFR-Akt signaling. A better understanding of the microenvironment that triggers invadosome formation and the signaling pathways involved will enable us to better understand the contribution of fibroblasts to the pathophysiology of IPF.

Our results indicate that hypoxia stimulates invadosome formation by normal mouse and human lung fibroblasts, as well as by the human HFL-1 cell line. These findings are in line with multiple studies demonstrating that hypoxia stimulates invadosome formation in cancer cells [22,42,43]. Moreover, gene expression of the invadosomal marker TKS5 was also induced upon exposure to hypoxia. We have previously demonstrated that TKS5 was predominantly expressed in IPF fibroblasts and the collagen-rich region of IPF lungs, where TKS5 expression is correlated with the ability of fibroblasts to form invadosomes [23]. Recently, TKS5-deficient mice were shown to be protected against bleomycin-induced pulmonary fibrosis [24]. We and others have found that the pro-fibrotic factors PDGF [23] and TGFβ [24,44] stimulate invadosome formation by lung fibroblasts. The induction of TKS5 expression by hypoxia is consistent with the previously reported increase in TKS5 gene expression in IPF lung tissue [23]. Altogether, these results indicate that hypoxia, a feature of IPF lungs, promotes invadosome formation.

Interestingly, hypoxia had no effect on invadosome formation in fibroblasts isolated from bleomycin-exposed mice (BLM) lungs. This is coherent with a previous report indicating that lung fibroblasts isolated from IPF patients express a persistent hypoxic signature compared with fibroblasts isolated from healthy tissue, and that incubation in a low-oxygen environment does not further activate hypoxia-dependent gene expression [33].

In addition to stabilizing the HIF-1 transcription factor, hypoxia is known to potentiate several signaling pathways, including the LPA-LPA_1_ axis [45]. In cancer cells, hypoxic induction of invadosome formation has been shown to involve LPA receptor signaling [17,22]. LPA_1_ is the main LPA receptor expressed by pulmonary fibroblasts, and the LPA-LPA_1_ axis was shown to be involved in pulmonary fibroblast migration and resistance to apoptosis [46]. LPA_1_ antagonists are in clinical trials for the treatment of IPF [36]. In the current study, LPA_1_ activation by LPA induced the formation of invadosomes by murine and human lung fibroblasts. Furthermore, inhibition of LPA_1_ in these fibroblasts prevented the hypoxic induction of invadosome formation, supporting the clinical relevance of LPA_1_ antagonists. These results suggest that LPA_1_ is essential for hypoxia to induce invadosome formation in healthy lung fibroblasts, consistent with previous findings in MDA-MB-231, HT1080, and U87 cancer cells [22].

Autotaxin (E-NPP 2) is a secreted phosphodiesterase that converts lysophosphatidylcholine (LPC) into LPA [47], and previous studies have reported that hypoxia increases LPA_1_ expression and autotaxin activity [17,22,48]. Thus, we wondered whether these mechanisms could explain the involvement of LPA axis in invadosome formation under hypoxic conditions in lung fibroblasts. We found that hypoxia had no effect on LPA_1_ mRNA and protein expression, as well as autotaxin mRNA expression and secretion in the supernatant of pulmonary fibroblasts. This result is consistent with a previous study showing, in the embryonic cerebral cortex, that hypoxia induces mitotic cell movement via LPA_1_, but hypoxia has no effect on LPA_1_ or autotaxin levels [48]. Further studies would be needed to assess the potential effect of hypoxia on other molecules involved in LPA regulation such as the lipid phosphate phosphatases (LPPs), which are responsible for the degradation of LPA to monoacylglycerol (MAG). Indeed, a recent study showed that hypoxia shifts the localization of LPP towards the trailing edge of cancer cell, while autotaxin is less diffuse and redistributed towards the leading edge [17]. Thus, hypoxia-induced LPP relocation may also promote the formation of an LPA gradient that would favor invadosome formation in lung fibroblasts.

Interestingly, it has been shown that hypoxia can also potentiate the transactivation of receptor tyrosine kinases (RTKs) by LPA_1_. In hypoxic HT1080 cells, EGFR was found to be activated by LPA_1_, which stimulates invadosome formation via the PI3K pathway [22]. In addition, the combined addition of EGFR and LPA_1_ inhibitors impaired metastasis in an ex vivo CAM model [22]. Among the RTKs, PDGFR has been shown to be phosphorylated on several tyrosine residues by hypoxia, in part through the production of ROS [49,50]. Recently, we demonstrated in lung fibroblasts that PDGFR activation by PDGF-BB induced the formation of invadosomes, a process that was also impaired by the addition of RTK and AKT inhibitors [23]. Given the importance of PDGFR and LPA_1_ in the profibrotic phenotype of IPF fibroblasts, we wondered whether the hypoxic induction of invadosome formation by pulmonary fibroblasts might involve LPA_1_-dependant PDGFR activation. Our results revealed that PDGFRβ is phosphorylated at residues Y857 and Y751 in pulmonary fibroblasts exposed to hypoxia. When phosphorylated, tyrosine 857 was shown to participate in the autophosphorylation of the receptor, a necessary step in its activation [51]. Tyrosine 751, on the other hand, can provide an anchoring site for PI3K, favoring the activation of the PI3K-Akt pathway [52]. These results suggest the involvement of PI3K-Akt in the hypoxic activation of PDGFRβ. We further showed that PDGFRβ and Akt phosphorylation was abolished by the addition of nintedanib as well as by LPA_1_ antagonists. Altogether, these results suggest that hypoxia activates the PDGFRβ-Akt signaling pathway likely with the participation of LPA_1_.

In conclusion, our study provides evidence that in lung fibroblasts, hypoxia-induced cell signaling is sufficient to induce the formation of invadosomes, cellular structures involved in matrix degradation and cell migration that were strongly associated with the severity of lung fibrosis. Lung hypoxia and increased invadosome formation by lung fibroblasts are characteristic features of IPF. Our results suggest that the hypoxia-induced invadosome formation in lung fibroblasts involves LPA_1_, as well as signaling through the PDGFRβ-Akt axis, as it could be suppressed by both LPA_1_ inhibition and the RTK inhibitor nintedanib. Because invadosome formation in lung fibroblasts is strongly associated with the severity of pulmonary fibrosis, targeting the hypoxic induction of invadosomes with combined LPA_1_ and RTK inhibitors may benefit patients with IPF.

## Figures and Tables

**Table 1:** Primer sequences.

Target Genes	Primer Sequences
*SH3PXD2A*	Forward: 5′-TGCCAAGAAGGAGATCAGCC-3′ Reverse: 5′-TGGAGGTCTTGTCCGTAGGT-3′
*RPL13*	Forward: 5′-CTCAAGGTCGTGCGTCTG-3′ Reverse: 5′-TGGCTTTCTCTTTCCTCTTCTC-3′
*CAIX*	Forward: 5′-CCTCCATAGCGCCAATGACT-3′ Reverse: 5′-CCTCAAGAACCCCAGATTATTGC-3′
*ENPP2*	Forward: 5′-GGGAGACCACGGATTTGATAA-3′ Reverse: 5′-ATCCCAGGAGATCACACATAAC-3′
*LPAR1*	Forward: 5′-AATCGAGAGGCACATTACGG-3′ Reverse: 5′-TGTGGACAGCACACGTCTAG-3′

## Data Availability

The data presented in this study are available on request from the corresponding author.

## Supplementary Materials

The following supporting information can be downloaded at https://www.mdpi.com/article/10.3390/cells13131152/s1, Figure S1: Hypoxia did not modulate LPA_1_ and autotaxin expression levels in lung fibroblasts.
