# Supplementary material for: Hypoxia Promotes Invadosome Formation by Lung Fibroblasts
Source: Cells. 2024 Jul 6;13(13):1152. doi: 10.3390/cells13131152 (PMC11240699; doi:10.3390/cells13131152)
Supplement: Supplementary file 1 [file cells-13-01152-s001.zip › cells-3094233-supplementary.pdf]

Lebel, M., et al., Hypoxia promotes Invadosome Formation by Lung Fibroblasts

Supplementary materials

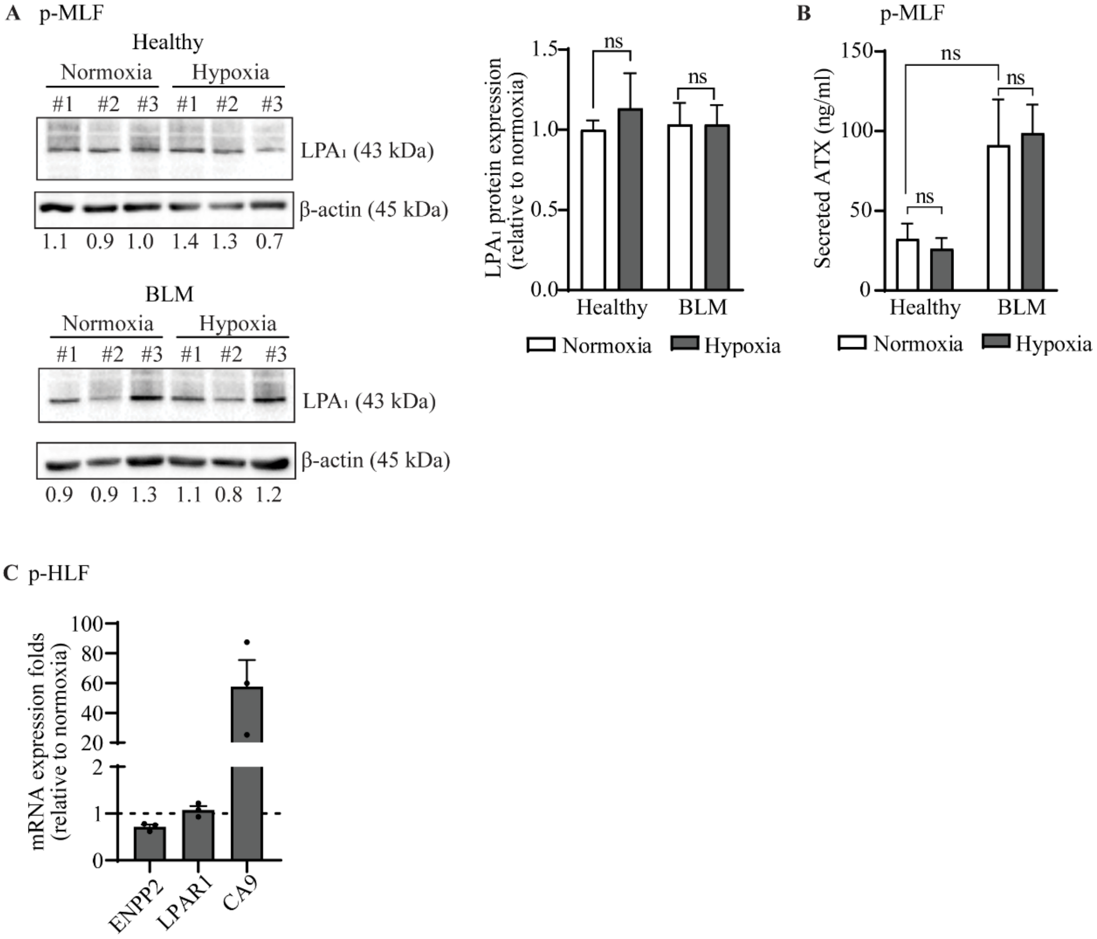

Figure S1. Hypoxia did not modulate LPA_1 and autotaxin expression levels in lung fibroblasts. Lung fibroblasts from healthy or BLM-exposed mice were incubated for 40h under hypoxia. **A)** Immunoblot of LPA_1 and β-actin (n = 3) with corresponding densitometric analysis relative to normoxia. **B**) Extracellular autotaxin assay normalized per mg of protein in lung fibroblast cell lysates (n = 5). **C**) The mRNA levels of LPA_1 (LPAR1), autotaxin (ENPP2) and the hypoxic positive control CAIX (CA9) were determined in primary human lung fibroblasts (p-HLF) incubated in hypoxia for 24 h and expressed in fold relative to normoxia. Each dot represents a cell culture from one healthy individual (n = 3). ns = not significant.
